# Deep learning software and revised 2D model to segment bone in micro-CT scans

**DOI:** 10.3389/fbinf.2025.1677527

**Published:** 2026-01-21

**Authors:** Andrew H. Lee, Ganesh Talluri, Manan Damani, Brandon Vera Covarrubias, Helena Hanna, Jeremy Chavez, Julian M. Moore, Jacob Baradarian, Michael Molgaard, Beau Nielson, Kalah Walden, Thomas L. Broderick, Layla Al-Nakkash

**Affiliations:** 1 Department of Anatomy, College of Graduate Studies, Midwestern University, Glendale, AZ, United States; 2 Arizona College of Osteopathic Medicine, Midwestern University, Glendale, AZ, United States; 3 College of Veterinary Medicine, Midwestern University, Glendale, AZ, United States; 4 Core Facilities-Glendale, Midwestern University, Glendale, AZ, United States; 5 BASIS Peoria, Peoria, AZ, United States; 6 Department of Physiology, College of Graduate Studies, Midwestern University, Glendale, AZ, United States

**Keywords:** artificial intelligence, avizo, bone, bone marrow, mammal, semantic segmentation

## Abstract

Deep learning (DL) enables automated bone segmentation in micro-CT datasets but can struggle to generalize across developmental stages, anatomical regions, and imaging conditions. We present BP-2D-03, which is a revised 2D Bone-Pores segmentation model. It was fitted to a dataset comprising 20 micro-CT scans spanning five mammalian species and 142,960 image patches. To manage the substantially larger and more varied dataset, we developed a DL software interface with modules for training (“BONe DLFit”), prediction (“BONe DLPred”), and evaluation (“BONe IoU”). These tools resolve prior issues such as slice-level data leakage, high memory usage, and limited multi-GPU support. Model performance was evaluated through three analyses. First, 5-fold cross-validation with three seeds per fold evaluated baseline robustness and stability. The model showed generally high mean Intersection-over-Union (IoU) with minimal variation across seeds, but performance varied more across folds related to differences in scan composition. These findings show that the baseline model is stable overall but that predictivity can decline for atypical scans. Second, 30 benchmarking experiments tested how model architecture, encoder backbone, and patch size influence segmentation IoU and computational efficiency. U-Net and UNet++ architectures with simple convolutional backbones (e.g., ResNet-18) achieved the highest IoU values, approaching 0.97. Third, cross-platform experiments confirmed that results are consistent across hardware configurations, operating systems, and implementations (Avizo 3D and standalone). Together, these analyses demonstrate that the BONe DL software delivers robust baseline performance and reproducible results across platforms.

## 1 Introduction

Deep learning (DL) models have emerged as powerful tools for automating bone segmentation in high-resolution micro-CT scans (e.g., Yu et al., 2022; Lee et al., 2025; Masuda et al., 2025). In a previous study, we demonstrated the utility of 2D and 3D convolutional neural networks as implemented in the commercial software Avizo 3D for distinguishing bone and medullary pores in long bones of North American river otters (*Lontra canadensis*) (Lee et al., 2025). Our results showed that both 2D and 3D models could achieve high segmentation performance when applied to skeletally mature bones, with mean Intersection over Union (IoU) scores exceeding 0.95 for bone and 0.94 for medullary pores. However, that study also highlighted three key limitations with the DL pipeline. First, it relied on a memory-intensive concatenation process to assemble the dataset, which limited scalability due to high system memory usage. Second, it performed slice-level rather than scan-level data partitioning, which introduced data leakage (i.e., adjacent slices from the same scan appeared in both training and validation sets). This likely led to optimistically biased estimates of performance and generalization because the models were partially evaluated on data that were not truly independent. Finally, the study did not implement a formal cross-validation framework (e.g., Bradshaw et al., 2023). Instead of repeatedly evaluating the model across several randomized scan-level partitions, performance was assessed on a single train-validation-test split. Consequently, the consistency and robustness of the model generalization across different subsets of the data remain unquantified.

**FIGURE 1:**
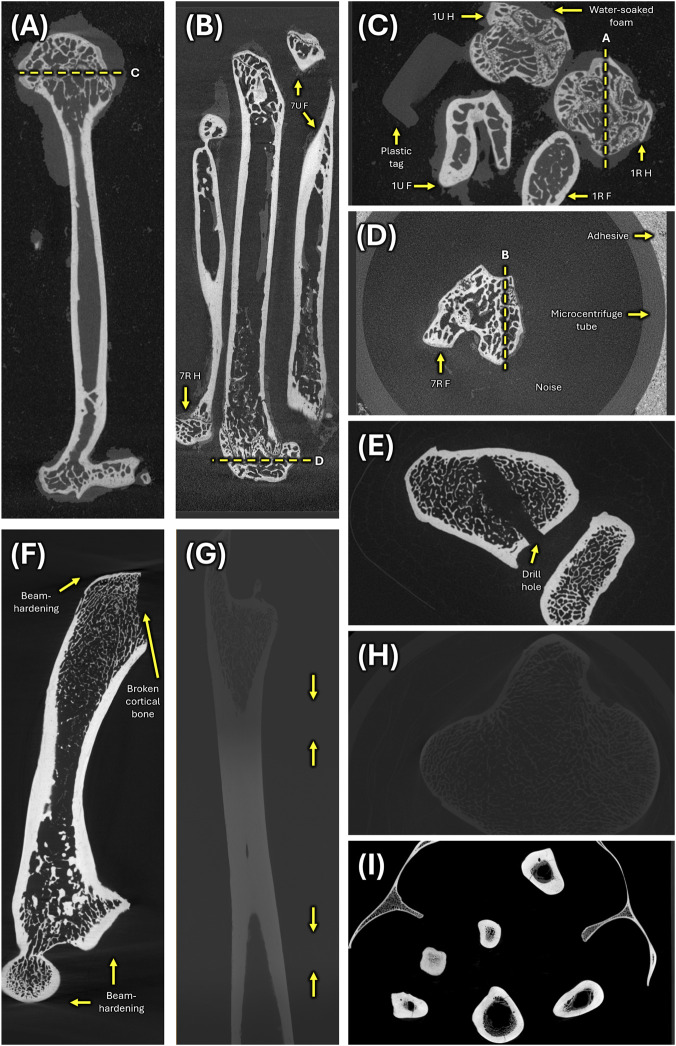
Variation in micro-CT scan quality and specimen condition of deep learning sample. Fully hydrated humeri and femora as seen in longitudinal **(A)** and transverse **(C)** views of mouse scan “1R_1U_HF.” An unanticipated delay in scanning of the rest of mouse sample resulted in dehydrated humeri and femora with contracted bone marrow as seen in longitudinal **(B)** and transverse **(D)** views of mouse scan “7R_7U_HF.” **(E)** River otter scan “UF_Mammals_31151_HRU” shows a humerus with deep artificial drill holes in the proximal and distal ends that were made by museum preparators to expedite degreasing. **(F)** Another river otter scan “UF_Mammals_23593–24550_HF” contains a humerus with a broken humeral head with exposed trabecular bone. Additionally, there are beam-hardening artifacts at the proximal and distal ends. **(G)** The leopard scan “AMNH_Mammals_M-89009_F” is three-part composite micro-CT scan with arrows highlighting the transitions between intensity domains. **(H)** The sea otter scan “ZMB_Mam_30740_HRU” is extremely dim with reduced contrast between bone and background. **(I)** The capybara scan “AMNH_Mammals_M-206440” contains limb, girdle and vertebral elements and was acquired at 8-bit instead of 16-bit depth, limiting how well subtle details can be distinguished.

Here, we address those limitations with updated deep learning software and present a revised 2D Bone-Pores (BP) segmentation model. We prioritized 2D models in this follow-up study because they showed slightly better segmentation performance (i.e., IoU score) and required less computational resources [i.e., system memory (RAM) and graphics processing unit memory (GPU VRAM)] than their 3D counterparts (e.g., Crespi et al., 2022; Lee et al., 2025). This software also enabled us to increase the size and variety of the deep learning dataset. In addition to 11 scans from the river otter sample that were used by Lee et al. (2025), we added nine scans from capybara, leopard, sea otter, and laboratory mouse (Table 1). The increased dataset covered a broad range of scanning resolution, imaging quality, and skeletal variation (Figure 1). Moreover, the inclusion of mouse bones to the dataset enabled the model to learn how to segment epiphyseal (growth) plates, which in mice are retained well into adulthood despite cessation of longitudinal bone growth (Roach et al., 2003).

**TABLE 1:** Properties of scans included in the deep learning sample.

Scan ID	Bones	2D Tiles	Voxel size (µm)	Source
1R 1U	HF	1,792	11.3	1
2R 2U	HF	2,112	9.1	
5R 5U	HF	2,048	9.1	
7R 7U	HF	2,048	9.1	
12R 12U	HF	2,048	9.1	
19R 19U	HF	1,920	9.1	
AMNH:Mammals:M-89009	H	4,250	66.8	2
AMNH:Mammals:M-206440	Mixed	1,672	120.7	3
OMNH:Mammals:44262	HRU	1,662	50.0	4
OMNH:Mammals:53994	FTFi	2,216	50.0	
OMNH:Mammals:53994	HRU	1,809	50.0	
UAM:Mamm:24789	FTFi	2,098	50.0	
UAM:Mamm:67696	HF	1,623	50.0	
UAM:Mamm:67696	TFiRU	2,321	50.0	
UF:Mammals:23593 UF:Mammals:24550	HF	1,755	50.0	
UF:Mammals:31151	HRU	1,660	50.0	
UWBM:Mamm:78743	FTFi	2,150	50.0	
UWBM:Mamm:81969	FTFi	2,195	50.0	
UWBM:Mamm:81969	HRU	1,995	50.0	
ZMB:Mam:30740	HRU	3,609	30.0	5

Bone abbreviations: F = femur; Fi = fibula; H = humerus; R = radius; T = tibia; U = ulna.

Museum abbreviations: AMNH, American Museum of Natural History; OMNH, Sam Noble Oklahoma Museum of Natural History; UAM, University of Alaska Museum of the North; UF, Florida Museum of Natural History; UWBM, University of Washington, Burke Museum; ZMB, Museum für Naturkunde.

Source abbreviations: 1 = doi. org/10.5061/dryad.4j0zpc8qq; 2 = ark:/87602/m4/430024; 3 = ark:/87602/m4/598442; 4 = doi. org/10.5061/dryad.b2rbnzsq4; 5 = ark:/87602/m4/M70721.

The software was written in Python to support streamlined model fitting and prediction (inference) while overcoming the memory inefficiencies of the previous pipeline. Furthermore, it enabled a systematic evaluation of three key model fitting choices: model architecture, encoder backbone, and patch size. Prior work has shown that these factors substantially influence segmentation quality across diverse biomedical applications (e.g., Yu et al., 2022; Ahmad et al., 2023; Masuda et al., 2025).

We examined four widely used architectures for semantic segmentation: U-Net (Ronneberger et al., 2015), UNet++ (Zhou et al., 2018), DeepLabV3+ (Chen et al., 2018), and SegFormer (Xie et al., 2021). These models differ in their strategies for balancing spatial resolution and feature abstraction. For example, U-Net and UNet++ rely on encoder-decoder designs with skip connections to preserve image details, whereas DeepLabV3+ uses atrous convolutions and a lightweight decoder to analyze features at multiple scales. SegFormer, in contrast, uses transformer-based attention mechanisms to capture long-range spatial dependencies.

To further explore how feature extraction affects segmentation IoU, we paired each architecture with one of four encoder backbones: ResNet-18, ResNet-50 (He et al., 2016), EfficientNet-B3 (Tan and Le, 2019), and MiT-B1 (Xie et al., 2021). These backbones vary in depth, GPU utilization, and ability to capture contextual information: ResNet encoders rely on convolutional residual blocks; EfficientNet-B3 employs compound scaling; and MiT-B1 uses attention-based operations derived from transformer networks.

**FIGURE 2:**
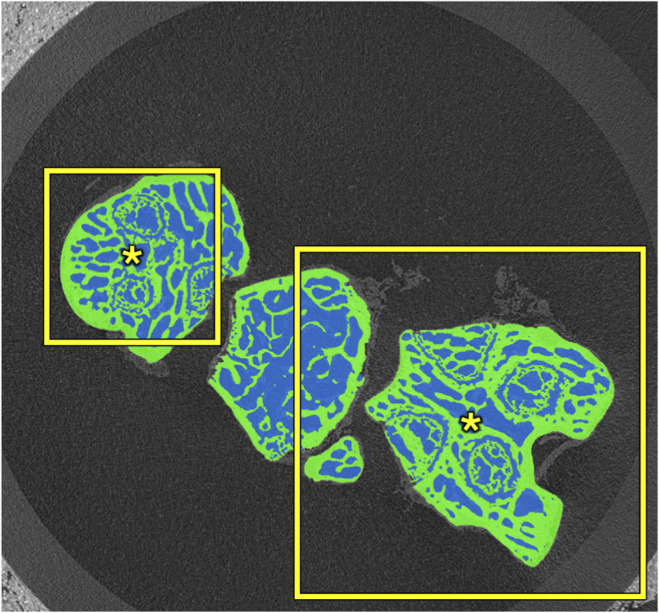
Trade-offs in patch size. Smaller patches (256 × 256 px) require less computation but may limit a model’s receptive field. The model may miss the full bone profile (green) and mislabel the background between closely-packed bones as medullary pores (blue). Larger patches (512 × 512 px) increase computational cost but expand the receptive field and may allow the model to learn broader structural patterns. The asterisks illustrate the relative centers from which the receptive field of each patch extends.

Finally, we compared two patch sizes (256 px and 512 px) to evaluate the tradeoff between local detail and broad spatial context. Our previous 2D model used 256-px patches (Lee et al., 2025), which provided a sufficient receptive field for identifying most boundaries between bone and medullary pores. However, a larger field of view could be important when distinguishing pores from background space between tightly-packed bones (Figure 2). By incorporating larger patches in the current study, we tested whether giving the model access to a wider receptive field improves model predictivity, while still maintaining reasonable computational costs.

This study aims to advance bone segmentation from micro-CT data by optimizing deep learning architectures, encoder backbones, and patch sizes for mammalian long bones. To support this effort, we developed an accessible, flexible, and memory-efficient software interface for model training, prediction, and evaluation. Designed for scalability, the pipeline is broadly applicable to skeletal imaging datasets and enables reproducible, high-quality segmentation across anatomical and biomedical research.

## 2 Materials and methods

### 2.1 Dataset collection

**FIGURE 3:**
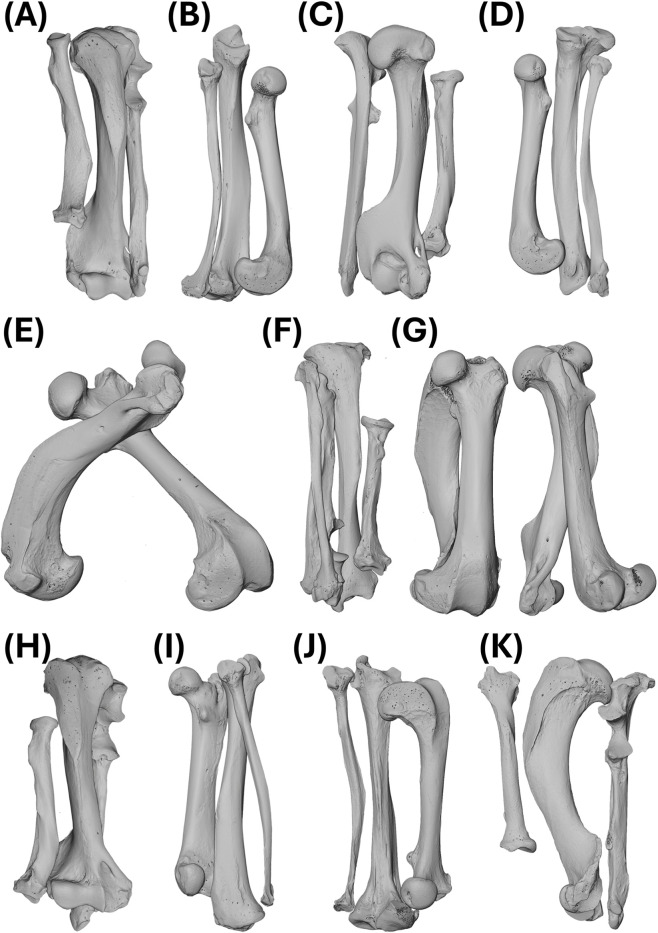
Volume rendering of scans from the North American river otter (*Lontra canadensis*). **(A)** OMNH_Mammals_44262_HRU: right humerus, left radius, and left ulna. **(B)** OMNH_Mammals_53994_FTFi: left femur, fibula, and tibia. **(C)** OMNH_Mammals_53994_HRU: left humerus, right radius, and right ulna. **(D)** UAM_Mamm_24789_FTFi: right femur, fibula, and tibia. **(E)** UAM_Mamm_67696_HF: left femur and humerus. **(F)** UAM_Mamm_67696_TFiRU: left fibula, radius, tibia, and ulna. **(G)** UF_Mammals_23593_HF and UF_Mammals_24550_HF: left femur and humerus. **(H)** UF_Mammals_31151_HRU: right humerus, radius, and ulna. **(I)** UWBM_Mamm_78743_FTFi: right femur, left fibula, and left tibia. **(J)** UWBM_Mamm_81969_FTFi: right femur, left fibula, and left tibia. **(K)** UWBM_Mamm_81969_HRU: left humerus, right radius, and right ulna. Abbreviations defined in Table 1. Not to scale.

**FIGURE 4:**
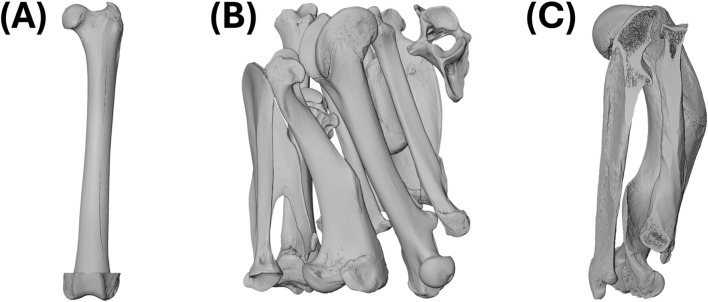
Volume rendering of scans from the leopard (*Panthera pardus*), capybara (*Hydrochoerus hydrochaeris*), and sea otter (*Enhydra lutris*). **(A)** AMNH_Mammals_M-89009_F: left femur of leopard. **(B)** AMNH_Mammals_M-206440_mixed: atlas; left calcaneus, femur, humerus, scapula, talus, and tibia; right calcaneus, radius, scapula, talus, and ulna. **(C)** ZMB_Mamm_30740_HRU: right humerus, radius, and ulna. Abbreviations defined in Table 1. Not to scale.

The deep learning dataset was assembled from three sources (Table 1). First, we included 11 micro-CT scans of long bones from the North American river otter (*Lontra canadensis*) (Figure 3) that were previously analyzed by Lee et al. (2025). Second, we downloaded three scans of long bones from capybara (*Hydrochoerus hydrochaeris*; AMNH:Mammals:M-206440), leopard (*Panthera pardus*; AMNH:Mammals:M-89009), and sea otter (*Enhydra lutris*; ZMB:Mam:30740) from MorphoSource (Figure 4). Third, we collected six micro-CT scans from a sample of laboratory mouse (*Mus musculus*) that are described below.

Forty male C57BL/6 mice (4-week old) were purchased from Charles River Laboratory (Wilmington, MA, United States) and maintained for 25 weeks. The mice were euthanized via asphyxiation in 100% atmospheric CO_2_, immediately followed by surgical thoracotomy to induce pneumothorax. All animal care was conducted in accordance with established guidelines, and all protocols used were approved by Midwestern University’s Institutional Animal Care and Use Committee (IACUC #AZ-4205).

Following surgical dissection of internal organs, skin, and subcutaneous tissues, the fore- and hindlimbs were removed from the axial skeleton at the glenohumeral and acetabulofemoral joints, respectively. The limbs from each mouse were fixed in 10% neutral buffered formalin for 24 h. After fixation, the limbs were grossly debulked of skin and muscles. Further dissection of the limbs was performed under illuminated magnification to mitigate unintentional cuts to the osteochondral surfaces. Radiocarpal and tibiotarsal joints were cut to detach the manus and pes, respectively. The remaining long bones (humeri, radii, ulnae, femora, tibiae, and fibulae) were isolated by severing residual ligamentous attachments. Any remaining non-skeletal tissue was gently removed with fine dissection tools. Dissected bone elements were rinsed with deionized (DI) water and stored in 70% ethanol.

**FIGURE 5:**
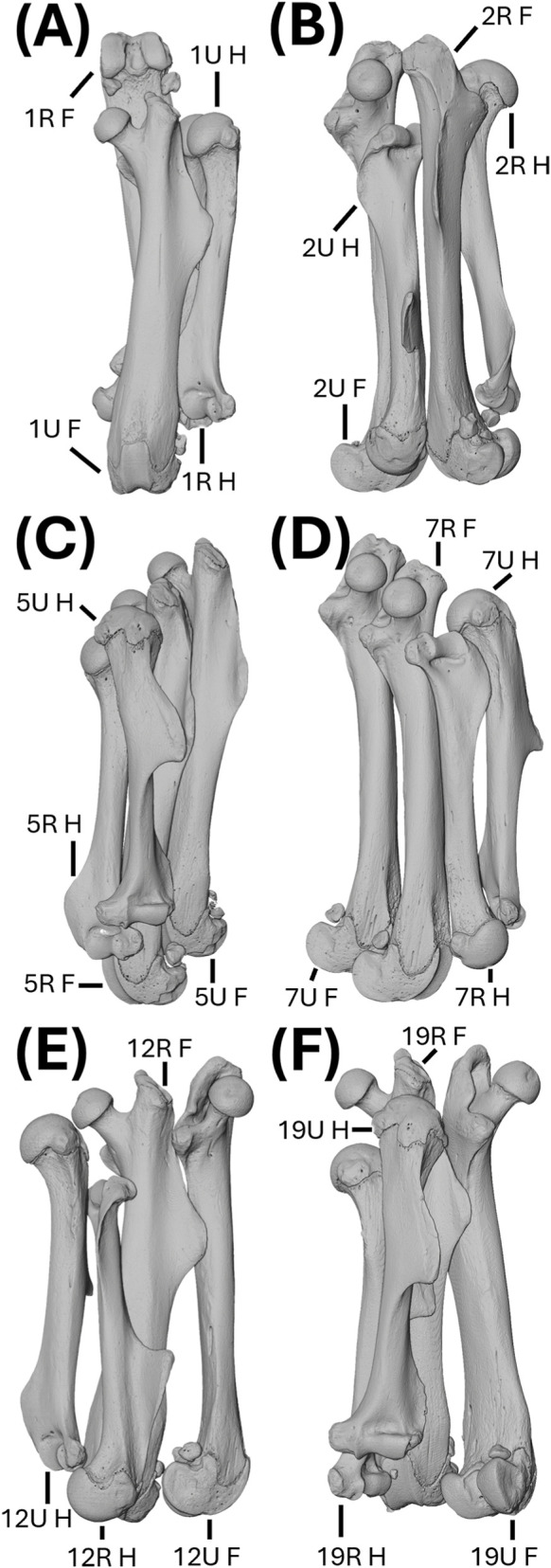
Volume rendering of scans from 12 adult laboratory mice (*Mus musculus*). **(A–F)** Scans: 1R_1U_HF; 2R_2U_HF; 5R_5U_HF; 7R_7U_HF; 12R_12U_HF; and 19R_19U_HF. Abbreviations defined in Table 1. Not to scale.

A subset of elements was selected for micro-CT scanning. The left humerus and femur from each mouse were rinsed with DI water and wrapped with melamine foam (Mr. Clean Magic Eraser, Procter and Gamble, Cincinnati, OH, United States). Twenty 1.5-mL microcentrifuge tubes (Thermo Fisher Scientific, Waltham, MA, United States) were prepared, and the bones from two mice were inserted into each tube. Micro-CT scanning was performed on a Nikon XT H 225 ST (Nikon Metrology Inc., Brighton, MI, United States) with settings at 120–160 kV, 58–112 μA, and 9.1–11.3 µm isotropic voxel size (Table 1). Only six out of the 20 scans were included in the current deep learning dataset. Because each scan contained the left humerus and femur from two individuals, this subset represents a total of 12 mice (Figure 5).

### 2.2 Preparing the reference masks

The micro-CT scans were imported into Avizo 3D 2024.2 (Thermo Fisher Scientific, Waltham, MA, United States). Bone-pores (BP) reference masks for these scans were segmented by the lead author following a previously published protocol (Lee et al., 2025). Briefly, bone tissue was initially segmented using the “Auto Thresholding” module followed by manual correction. Thin trabeculae that were not captured by thresholding were recovered using the “white top hat” filter. Cortical and medullary pores were segmented using the “Compute Ambient Occlusion” module. Note: the ambient occlusion algorithm tended to mislabel background voxels as pore voxels in deep concavities such the coronoid, olecranon, and intertrochanteric fossae (Bab et al., 2007b; 2007a) and required manual correction. For those areas, we used a standard thresholding value of 0.95 to ensure that deep concavities were segmented consistently across reference masks.

### 2.3 Three deep learning modules for Avizo

#### 2.3.1 “BONe DLFit”

**FIGURE 6:**
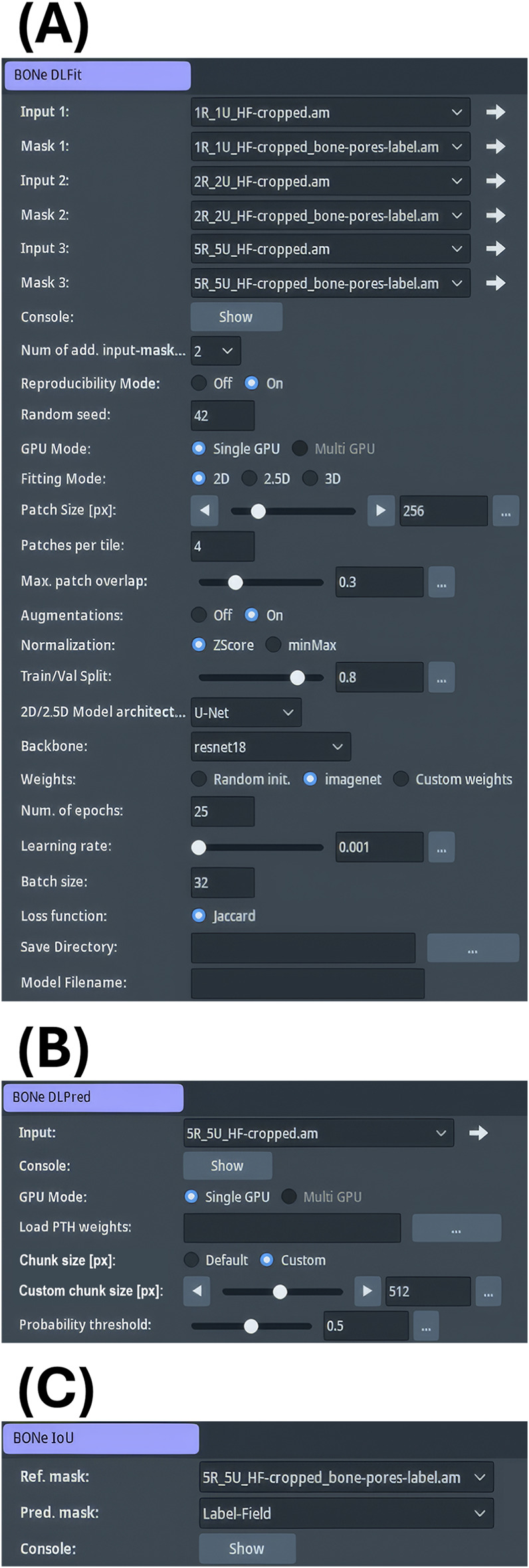
Graphical interface of deep learning modules in Avizo 3D 2024.2. **(A)** “BONe DLFit” provides point-and-click fields for up to 20 scan-mask pairs, along with additional options for fitting segmentation models. **(B)** “BONe DLPred” applies the selected PyTorch model to an input scan and allows users to set the chunk size. This does not alter the model’s receptive field; instead, it divides a large scan into smaller chunks that are more easily fit into GPU memory. **(C)** “BONe IoU” calculates class-wise and mean IoU between reference and prediction masks.

We developed a module to fit 2D deep learning (DL) models in Avizo 3D 2024.2 (Figure 6A) that overcomes several limitations of Avizo’s built-in “DL - Segmentation 2D” module. First, the built-in module only enables a single pair of scan-mask connection ports. To train the computer on multiple pairs, users must concatenate all scans into one large volume and all masks into another, which requires padding them to the same XY dimensions, greatly increasing memory demands and limiting sample size. Our custom module supports up to 20 scan-mask pairs via connection ports, eliminating the need for concatenation or padding. Note: the source code may be altered to support more than 20 pairs of input ports. However, this necessitates additional scrolling in the graphical interface. Second, the built-in module allows data leakage between the training and validation set, resulting in optimistically biased model performance. The custom module addressed the data leakage problem by performing the training-validation split at the scan-level. Third, the built-in module is limited to a single GPU, which restricts batch size and parallelization. The custom module gives the user the option to use multiple CUDA-compatible GPUs if available via PyTorch’s “DataParallel” library (version 2.8.0+cu129: Paszke et al., 2019). Fourth, the built-in module only supports the U-Net architecture (Ronneberger et al., 2015) with three pre-defined backbones. In contrast, our module allows users to select from nine model architectures and 58 backbones via the “segmentation_models_pytorch” library (version 0.5.0: Iakubovskii, 2019). Finally, the built-in module is difficult to customize because the core code is compiled. The custom module is uncompiled, and advanced users are free to further customize and extend it.

“BONe DLFit” features a graphical frontend with a point-and-click interface (Figure 6A), making DL model fitting accessible to non-expert users. The backend uses PyTorch (Paszke et al., 2019) for efficient training and evaluation of deep learning models, which consists of a pipeline with three major stages: (1) collecting user options, (2) initialization of the DL pipeline, and (3) the model fitting loop.

**FIGURE 7:**
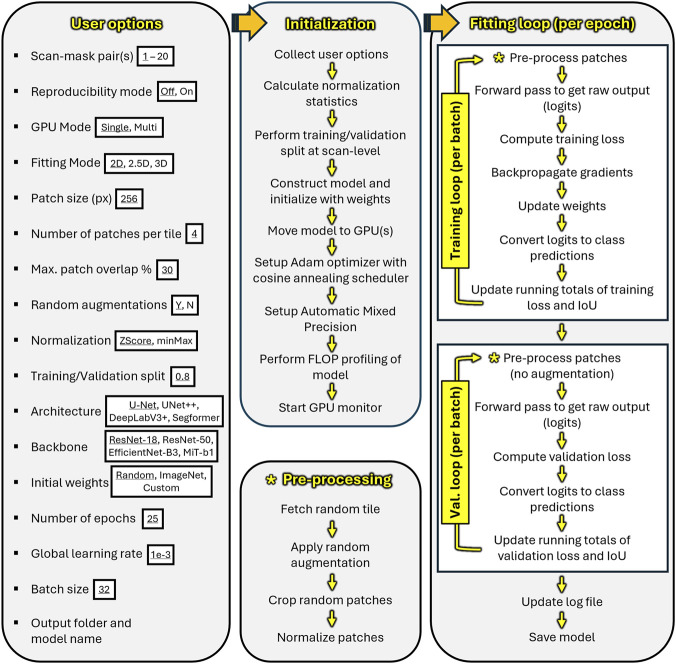
Schematic of the model fitting pipeline implemented by “BONe DLFit.” (Left) User options. (Center) The module collects user options, calculates normalization statistics, splits the dataset at the scan-level, initializes the fitting loop, profiles model complexity, and launches a GPU monitor. (Right) Training proceeds in batches with image augmentation, forward pass, loss computation (Jaccard loss), backpropagation, and weight updates. Validation proceeds similarly but without augmentation. Metrics such as loss and Intersection-over-Union (IoU) are accumulated and logged. Model weights are saved when validation performance improves, and the global learning rate is adjusted via cosine annealing.

In the first stage, “BONe DLFit” records the user-specified configuration to define the data handling, model architecture, and training procedure (Figure 7). These options include:

- Input source(s): supports up to 20 scan-mask input pairs.
- Reproducibility mode: fixes random seeds and enables deterministic algorithms.
- GPU mode: toggles single or multi-GPU support.
- Fitting mode: toggles support for 2D, 2.5D, or 3D models.
- Patch cropping: alters the patch size, number of random patches to crop per tile (tile is defined as a 2D slice or 3D slab), and maximum patch overlap.
- Data augmentation: toggles random transformations (e.g., flips, rotation in multiples of 90°, brightness, and contrast) on tiles.
- Normalization: toggles between Z-score and min-max normalization.
- Training/validation split: sets the fraction of whole scans used for training, while the remaining scans are reserved for validation to assess model performance.
- Model architecture: specifies the design of the neural network (e.g., U-Net, UNet++, DeepLabV3+, SegFormer).
- Backbone: defines the pattern extracting portion of the architecture (e.g., ResNet-18, EfficientNet-B3, MiT-B1).
- Initial weights: defines starting values of the model’s learnable parameters, which are either randomly initialized or transferred from a previously trained model (ImageNet-trained or user-provided).
- Hyperparameters: control speed and stability of training, specifically the number of epochs (i.e., the number of full passes through the training dataset), global learning rate (i.e., the step size used to update model weights), and batch size (i.e., the number of patches processed together in each update).
- Save location: saves the model and log files onto computer storage.

In the second stage, “BONe DLFit” calculates normalization statistics, initializes the user-specified model, and configures the DL pipeline (Figure 7). The statistics used for image normalization are pre-computed using CPU-based multiprocessing, which reduces wall time. Because multiprocessing inside Avizo unintentionally triggers additional license usage, the statistics are pre-computed using an external standalone Python interpreter (version 3.12.11), allowing worker processes to run independently of Avizo and without drawing extra licenses. Immediately following the calculation of normalization statistics, the scans are randomized and split at the scan-level into training and validation sets as closely as possible to the user-specified ratio. The selected architecture, backbone, and Jaccard loss function are imported from “segmentation_models_pytorch”, and the Jaccard index (Jaccard, 1912) (also known as Intersection-over-Union or IoU) is imported from “torchmetrics” (version 1.8.2: Detlefsen et al., 2022). User-specified initial weights are loaded onto the model. Depending on user choice, the model is prepared for single- or multi-GPU training using “DataParallel”. The Adam optimizer (Kingma and Ba, 2015) is configured with the user-defined global learning rate followed by the initialization of a single-cycle cosine annealing scheduler without restarts (e.g., Loshchilov and Hutter, 2017; He et al., 2019) to stabilize convergence. Automatic mixed precision is enabled via PyTorch’s “autocast” to switch automatically between less precise float16 and more precise float32 calculations for efficient memory usage (He et al., 2019). Model complexity is estimated based on counts of floating-point operations (FLOPs) and parameters via “FlopCountAnalysis” and “parameter_count_table” from the fvcore library (version 0.1.5. post20221221). Lastly, a GPU monitor is launched to track peak VRAM usage and GPU utilization during the fitting loop.

In the final stage, “BONe DLFit” enters the fitting loop, which repeats for each epoch (Figure 7). The training dataset is processed first. Matching 2D tiles from each scan-mask pair are randomly sampled without replacement. Optional augmentation is applied based on the following probabilities: horizontal flip (16.6%), vertical flip (16.6%), 90°, 180°, or 270° rotation (50%), or no augmentation (16.8%). Rotation is implemented in 90° increments to avoid introducing aliasing artifacts when augmenting the reference mask (Stone et al., 2003). Random patches are cropped from each scan-mask pair. After cropping, the scan patches are normalized using scan-specific statistics (either Z-score or min-max normalization). If augmentation is enabled, the normalized patches undergo intensity augmentation in which global brightness shifting and contrast rescaling are applied with independent probabilities (each 40%). Consequently, either augmentation may occur alone, both may occur together, or neither may occur. When selected, a single random shift value (uniformly sampled from −0.15 to 0.15) and a single random scaling factor (sampled from 0.75 to 1.25) are applied to all scan patches. These operations simulate variability in scanner calibration, illumination, or tissue contrast across datasets. Afterward, intensities are clipped to remain within the expected dynamic range of the chosen normalization mode. The resulting patches are assembled into batches and passed to the model for forward and backward propagation during training.

For each batch, Jaccard loss and gradients are computed, and model weights are updated immediately. Jaccard loss and IoU scores are then aggregated across all batches within an epoch to produce stable and representative epoch-level measurements of model performance.

Once all the training data are processed, the model switches to validation mode, wherein the module assembles cropped patches from the validation data into batches without augmentation before passing them to the model for evaluation. Validation loss and IoU are computed, and accumulated results are updated. Each epoch concludes with three steps: (1) updating the log file, (2) saving model weights in PTH file format upon improvement in validation performance compared to the previous epoch, and (3) reducing the global learning rate following a cosine-curve scheduler to allow for smoother convergence.

#### 2.3.2 “BONE DLPred”

**FIGURE 8:**
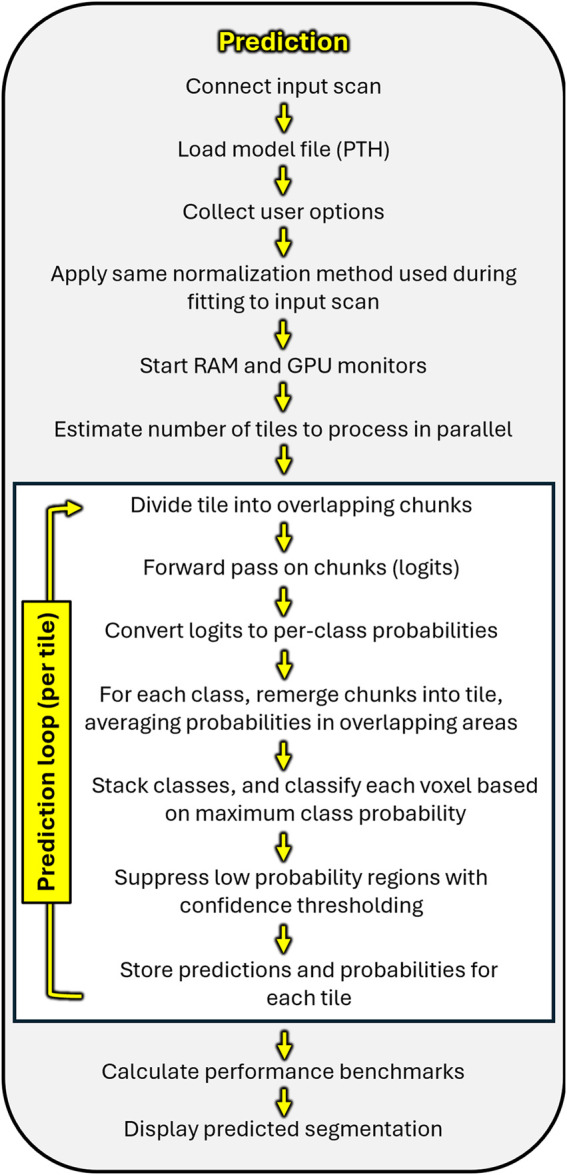
Schematic of the prediction (inference) pipeline implemented by “BONe DLPred.” The module accepts user inputs including the scan to segment, model weights file, GPU mode, chunk size, and confidence threshold. After loading the model and applying the same normalization used during fitting, the input scan is divided into user-specified overlapping chunks. The chunks are processed by the model in batches, then reassembled by averaging probabilities in overlapping regions. Voxels are classified based on maximum class probability, with low-confidence areas suppressed via thresholding. Finally, performance benchmarks and the segmentation output are displayed.

Avizo includes a built-in inference module (“Deep Learning Prediction”), but it does not support models formatted for PyTorch. Therefore, we developed a module called “BONe DLPred”. The graphical frontend of the module accepts several input fields: (1) the input scan to segment, (2) the option for single- or multi-GPU inference, (3) the weights PTH file, (4) the chunk size for prediction, and (5) the confidence threshold (Figure 6B). After recording the user options, the backend loads the weights PTH file, which includes embedded information (metadata) about the model such as model type (2D, 2.5D, or 3D), architecture, backbone, hyperparameters, and method of image normalization used during model fitting. RAM and GPU monitors are launched for benchmarking purposes. Lastly before entering the prediction loop, the module estimates the number of tiles to process in parallel (Figure 8).

The module then enters the prediction loop, which processes the input scan in batches of tiles rather than one tile at a time. For each batch, overlapping chunks are extracted using a customized routine based on “empatches” (version 0.2.3: Ilyas, 2023). This customization supports 2D, 2.5D, and 3D inputs, and for 3D inputs, allows chunk depth to differ from chunk width and height. The chunks are normalized, passed through the model, and subsequently reassembled into a full-resolution probability map using overlap-aware merging. Class labels are assigned by selecting the maximum-probability class at each voxel, with user-defined confidence-based background reassignment. The reconstructed tiles are accumulated sequentially to form the final multi-class segmentation mask. Finally, confidence statistics and performance benchmarks are displayed (Figure 8).

#### 2.3.3 “BONe IoU”

Previously, we developed a tool command language (TCL) script within Avizo to calculate IoU Score, which quantifies the overlap between predicted and reference segmentation (Lee et al., 2025). Although functional, the script had several drawbacks. It computed IoU score one class at a time, did not automatically calculate the mean IoU score, cluttered the Avizo Project View with temporary data objects, and was relatively slow. To address these issues, we developed a Python module, “BONe IoU”, which automated the calculation of both class-wise and mean IoU scores with substantially improved speed and efficiency (Figure 6C).

After loading the user-specified reference and predicted segmentation masks, “BONe IoU” checks for the presence of a CUDA-compatible GPU. If one is available, it performs the IoU calculation using PyTorch tensors on the GPU. If not, the calculation defaults to using NumPy (version 1.23.5: Harris et al., 2020) arrays on the CPU.

#### 2.3.4 Standalone versions of “BONe DLFit”, “BONe DLPred”, and “BONe IoU”

To ensure accessibility for users without Avizo 3D, we developed standalone Python versions of all three modules. These versions preserve the names, graphical interfaces, and core functionality of their Avizo counterparts but run entirely outside the Avizo environment. Each module is packaged with an updated Python (3.12.11) and NumPy (2.3.4) backend while retaining the same PyTorch version (2.8.0+cu129) used by the Avizo 3D implementation. From the user’s perspective, the standalone apps operate nearly identically to the Avizo modules with the difference being the organization and format of the input scans and masks being folders of TIFF files. Nevertheless, models are interchangeable between them. Versioning is clearly indicated in the log files and weights files (e.g., “1.0.0. avizo” or “1.0.0. standalone”).

### 2.4 Computation

#### 2.4.1 Hardware specifications

Deep learning experiments were conducted on three workstations with varying performance capabilities to demonstrate that the Avizo 3D and standalone versions of the BONe apps function across both high-end and older hardware configurations (Table 2). “Jarvis” and “Hopper” are high-end workstations with the former configured with twice as much VRAM. Both are capable of dual-boot operation in Ubuntu 22.04 LTS (Canonical Ltd., London, England, United Kingdom) and Windows 11 Pro (Microsoft, Redmond, WA, United States). “Friday” is a more modest configuration running Windows 10 Pro. It was only used for prediction given its limited RAM and slow GPU performance.

**TABLE 2:** Hardware configurations.

Workstation	“Jarvis”	“Hopper”	“Friday”
OS	Dual boot: Ubuntu 22.04 LTS Windows 11 Pro	Dual boot: Ubuntu 22.04 LTS Windows 11 Pro	Windows 10 Pro
CPU	AMD Threadripper PRO 5965WX	AMD Threadripper PRO 7995WX	Intel i9-10900X
RAM	512 GB DDR4 3200 MT/s	512 GB DDR5 5200 MT/s	256 GB DDR4 2666 MT/s
GPU	2x Nvidia RTX PRO 6000 Blackwell Max-Q 96 GB	2x Nvidia RTX 6000 Ada 48 GB	Nvidia Quadro P6000 24 GB
Main storage	Samsung 990 2 TB M.2 NVMe PCIe 4.0	Kingston NV3 2 TB M.2 NVMe PCIe 4.0	Samsung 970 EVO 500 GB M.2 NVMe PCIe 3.0
Vendor	Author-assembled	Puget Systems	Titan Computers
Price (year)	$25,500 (2025)	$31,100 (2024)	$7,500 (2020)

#### 2.4.2 Performance comparison across platforms

To assess the reproducibility and stability of the BONe versions across different hardware and operating systems, we evaluated both fitting and prediction performance on the three workstation configurations described above (Table 2). For comparability, all platforms used the same dataset composition and model configuration.

Fitting performance was assessed using Training/Validation Pool 1 (Table 3) and a fixed random seed (seed 42). All experiments used the same baseline model configuration: U-Net with a ResNet-18 backbone, 2D fitting mode, and 256-px patch size. Each 2D tile (slice) produced four random patches, resulting in 120,520 training patches and 22,440 validation patches per epoch (scan-level split of 81.25:18.75). Data augmentation was enabled and included random flips, rotations in 90° increments, crops, and domain-shift transformations. Z-score normalization was performed on the patches. The model was initialized with ImageNet-trained weights, following common practice in medical imaging segmentation (e.g., Deng et al., 2009; Iglovikov and Shvets, 2018; Alzubaidi et al., 2021; Lösel et al., 2023). Training proceeded for 25 epochs using a batch size of 64, an initial global learning rate of 0.001 with cosine-annealing scheduling, Adam optimizer, Jaccard loss as the optimization objective, and IoU as the evaluation metric. Single and dual GPU operation was compared. Model fitting performance (e.g., GPU utilization, fitting time, and wall time) was recorded in Table 4 for each combination of workstation, operating system, BONe version, and GPU count.

**TABLE 3:** Overview of the 20 scans used for 5-fold cross-validation. The scans were first randomized and then placed into a fixed order prior to partitioning into test fold sets. For each Test Fold, the Training/Validation Pool comprised the remaining 16 scans in sequence order (e.g., Training/Validation Pool 1 consisted of scan number 5–20).

Order	Scan ID	Test fold
1	UF_Mammals_31151_HRU	1
2	OMNH_Mammals_44262_HRU	
3	2R_2U_HF	
4	OMNH_Mammals_53994_HRU	
5	UWBM_Mamm_81969_HRU	2
6	UWBM_Mamm_78743_FTFi	
7	12R_12U_HF	
8	AMNH_Mammals_M-206440_mixed	
9	OMNH_Mammals_53994_FTFi	3
10	UWBM_Mamm_81969_FTFi	
11	UF_Mammals_23593–24550_HF	
12	UAM_Mamm_67696_HF	
13	19R_19U_HF	4
14	1R_1U_HF	
15	AMNH_Mammals_M-89009_F	
16	7R_7U_HF	
17	UAM_Mamm_24789_FTFi	5
18	5R_5U_HF	
19	ZMB_Mam_30740_HRU	
20	UAM_Mamm_67696_TFiRU	

**TABLE 4:** Model fitting performance of “BONe DLFit” compared across platforms. Dataset used was Training/Validation Pool 1 with a random seed of 42.

Workstation	OS	BONe version	GPU #	GPU usage (%)	Peak VRAM (GB)	Peak RAM (GB)	Val. mIoU	Val. mDice	Wall Time (s)
“Jarvis”	L	S	2	84.0	22.5	419.8	0.9775	0.9886	9,140
	L	A	2	83.0	22.5	446.0	0.9790	0.9894	9,303
	L	S	1	99.4	23.1	440.2	0.9774	0.9886	14,852
	L	A	1	99.3	23.3	442.1	0.9778	0.9888	14,944
“Hopper”	L	A	2	89.5	22.7	446.8	0.9778	0.9888	9,862
	L	S	2	89.1	22.5	412.7	0.9774	0.9886	9,975
	L	A	1	99.5	20.0	447.0	0.9775	0.9886	17,289
	L	S	1	99.6	19.8	426.0	0.9783	0.9890	17,374
	W	S	2	38.6	22.7	366.1	0.9769	0.9883	22,796
	W	A	2	42.1	22.7	351.9	0.9760	0.9879	27,058
	W	S	1	58.7	20.0	367.7	0.9758	0.9878	28,871
	W	A	1	65.1	20.1	355.8	0.9736	0.9866	33,341

Abbreviations: A = Avizo 2024.2; S=Standalone; L = Ubuntu 22.04 LTS; W=Windows 11 Pro; Val = Validation.

Prediction performance was evaluated using a standardized model (BP-2D-03) that was fitted on the “Jarvis” workstation running Linux Avizo 3D 2024.2. The model was applied to mouse scan “2R_2U_HF,” which was one of the Test Fold 1 scans and not seen during training and validation. The chunk size was increased to 512 px to reduce visible seam lines during reassembly of the full-size output. Default confidence thresholding of 0.5 was applied so that low-confidence voxels were reassigned to background. Prediction-stage resource usage (GPU utilization, peak VRAM, peak RAM, and wall time) was recorded for each platform (Table 5).

**TABLE 5:** Cross-platform performance of “BONe DLPred” when segmenting scan “2R_2U_HF” using model BP-2D-03.

Workstation	OS	BONe version	GPU #	GPU usage (%)	Peak VRAM (GB)	Peak RAM (GB)	mIoU	mDice	Wall Time (s)
“Jarvis”	L	A	2	43.7	72.4	41.3	0.9829	0.9914	55
	L	A	1	60.5	42.9	43.3	0.9829	0.9914	60
	L	S	2	33.6	77.0	39.6	0.9829	0.9914	68
	L	S	1	53.8	41.0	42.3	0.9829	0.9914	75
“Hopper”	L	A	2	66.7	28.8	39.7	0.9829	0.9914	59
	L	S	2	36.3	28.1	37.1	0.9829	0.9914	62
	L	A	1	83.2	16.3	40.9	0.9829	0.9914	68
	L	S	1	63.8	14.6	37.6	0.9829	0.9914	71
	W	S	1	55.0	15.2	62.3	0.9829	0.9914	85
	W	S	2	39.9	28.2	60.0	0.9829	0.9914	85
	W	A	2	57.5	28.7	48.9	0.9829	0.9914	95
	W	A	1	72.0	13.8	50.6	0.9829	0.9914	102
“Friday”	W	A	1	79.8	12.0	37.5	0.9829	0.9914	277

Abbreviations: L = Ubuntu 22.04 LTS; W=Windows 10/11 Pro.

### 2.5 Model evaluation

#### 2.5.1 Cross-validation to assess model generalization and stability

Cross-validation experiments were performed on the “Jarvis” workstation, operating in dual-GPU mode in Linux Avizo 3D. We conducted 5-fold cross-validation using the 20 scans listed in Table 3. The scans were first randomized and assigned a fixed order prior to partitioning into five test-fold sets with each fold containing four scans and the remaining scans (in sequence order) forming a training/validation pool. To assess the stability of the results, we repeated the full 5-fold partitioning using three random seeds (42, 1701, and 1864), yielding a total of 15 models.

Model fitting was performed using the baseline configuration described in Section 2.4.2. The resulting model was applied to each of the four scans in the corresponding test fold, and mIoU scores were averaged (Supplementary Table S1). Cross-validation performance was summarized as grand mIoU across all folds and replicates (Table 6), which also reports the corresponding mDice values. A conversion of scan-level mIoU scores to mDice is provided in Supplementary Table S2.

**TABLE 6:** Summary of 5-fold cross-validation results. Each test fold was evaluated under three random seeds (42, 1701, 1864), with performance reported as mean Intersection over Union (mIoU ±SD). The grand mean IoU ±SD aggregated across folds and seeds is bolded and shown in the lower-right cell. Bracketed values are the mIoU results converted to mDice.

Test fold	mIoU (seed 42)	mIoU (seed 1701)	mIoU (seed 1864)	Mean ± SD across seeds
1	0.9731 ± 0.0099 [0.9864 ± 0.0051]	0.9738 ± 0.0106 [0.9867 ± 0.0054]	0.9723 ± 0.0099 [0.9860 ± 0.0051]	0.9731 ± 0.0092 [0.9863 ± 0.0047]
2	0.8463 ± 0.2501 [0.8992 ± 0.1724]	0.8533 ± 0.2287 [0.9066 ± 0.1538]	0.8523 ± 0.2309 [0.9057 ± 0.1557]	0.8506 ± 0.2142 [0.9038 ± 0.1455]
3	0.9420 ± 0.0376 [0.9699 ± 0.0201]	0.9301 ± 0.0497 [0.9633 ± 0.0270]	0.9456 ± 0.0298 [0.9719 ± 0.0158]	0.9393 ± 0.0367 [0.9683 ± 0.0198]
4	0.8883 ± 0.1438 [0.9358 ± 0.0875]	0.8913 ± 0.1488 [0.9372 ± 0.0907]	0.8508 ± 0.1740 [0.9115 ± 0.1121]	0.8768 ± 0.1425 [0.9282 ± 0.0889]
5	0.9542 ± 0.0395 [0.9763 ± 0.0211]	0.9264 ± 0.0939 [0.9599 ± 0.0533]	0.8995 ± 0.1492 [0.9418 ± 0.0902]	0.9267 ± 0.0972 [0.9593 ± 0.0577]
Mean ± SD across folds	0.9208 ± 0.0522 [0.9535 ± 0.0358]	0.9150 ± 0.0452 [0.9507 ± 0.0303]	0.9041 ± 0.0546 [0.9434 ± 0.0356]	**0.9133 ± 0.0476** **[0.9492 ± 0.0318]**

#### 2.5.2 Assessing the effect of model architecture, backbone, and patch size

Subsequent experiments evaluating the effects of architecture, backbone, and patch size were performed on the “Jarvis” workstation with Training/Validation Pool 1 (seed 42). This dataset provided the most favorable balance of high mIoU (0.9731) and low variability (SD = 0.0099), making it the most stable and representative among the available cross-validation splits. In total, we tested 30 combinations of architecture, backbone, and patch size (Supplementary Table S3). Training conditions were kept as consistent as possible across these combinations and followed the settings used in section 2.4.2. However, some UNet++, SegFormer, and MiT-B1 models required more VRAM than was available, so batch size was reduced from 64 to 32 (Supplementary Table S3). In addition, models with the MiT-B1 backbone and 512-px patches failed to converge under the default settings and therefore required the learning rate to be lowered from 1e-3 to 1e-4 (Supplementary Table S3). For completeness, Supplementary Table S4 reports the corresponding Dice scores (mean, bone, and pores) converted from the IoU values.

#### 2.5.3 Weighted scoring and ranking of models

For each of the 30 model combinations, mean, bone, and pores IoU scores were averaged, respectively, across the four scans of Test Fold 1 (Supplementary Table S3). To evaluate the trade-off between performance and computational efficiency, we calculated a weighted score for each model using the following normalized metrics:

$${mIoU}_{norm}=\frac{mIoU-{mIoU}_{\min}}{{mIoU}_{\max}-{mIoU}_{\min}},$$

$$B_{norm}=\frac{B-B_{\min}}{B_{\max}-B_{\min}},$$

$$U_{norm}=\frac{U-U_{\min}}{U_{\max}-U_{\min}},$$

$$F_{norm}=1-\frac{F-F_{\min}}{F_{\max}-F_{\min}},$$

$$P_{norm}=1-\frac{P-P_{\min}}{P_{\max}-P_{\min}},$$

$$V_{norm}=1-\frac{V-V_{\min}}{V_{\max}-V_{\min}},$$

$$T_{norm}=1-\frac{T-T_{\min}}{T_{\max}-T_{\min}}.$$

Here, *mIoU* denotes mean IoU from Test Fold 1, *B* is the batch size; *U* is average GPU utilization; *F* is the total number of floating-point operations (FLOPs) executed on a one-batch sample from the training set during a single forward pass of the network; *P* is the parameter count; *V* is the GPU VRAM consumed during fitting; and *T* is the time spent in the fitting loop. Higher values are preferrable for mean IoU, batch size, and GPU utilization, whereas lower values are preferrable for FLOPs, parameters, VRAM consumption, and fitting time. The weighted score of each model was then calculated as:

$$\begin{array}{ll} & weighted\;score=0.85\;{mIoU}_{norm}+0.025\;B_{norm}+0.025\;U_{norm}\\ & \;+0.025\;F_{norm}+0.025\;P_{norm}+0.025\;V_{norm}+0.025\;T_{norm}. \end{array}$$

The values of the weighting coefficients were selected to place a strong emphasis on model predictivity (weight = 0.85) while allocating the remaining 0.15 equally across the six complementary efficiency-related metrics. To assess the robustness of this scoring framework, we performed a sensitivity sweep by varying the relative weight assigned to mean IoU (Table 7). Across the tested weightings, top-ranked models remained largely consistent, indicating that the ranking procedure is stable with respect to reasonable changes in the weighting scheme.

**TABLE 7:** Sensitivity of model rankings to changes in performance–efficiency weighting.

mIoU weight	Efficiency weight per metric^a^	Top 3 models (weighted score)
1.00	0	1. UNet++ | EfficientNet-B3 | 256 px (1.0000) 2. U-Net | EfficientNet-B3 | 256 px (0.9994) 3. U-Net | ResNet-18 | 256 px (0.9934)
0.95	≈0.008	1. U-Net | ResNet-18 | 256 px (0.9913) 2. UNet++ | ResNet-18 | 256 px (0.9865) 3. UNet++ | EfficientNet-B3 | 256 px (0.9861)
0.90	≈0.017	1. U-Net | ResNet-18 | 256 px (0.9893) 2. UNet++ | ResNet-18 | 256 px (0.9833) 3. U-Net | ResNet-18 | 512 px (0.9730)
0.85	0.025	1. U-Net | ResNet-18 | 256 px (0.9873) 2. UNet++ | ResNet-18 | 256 px (0.9802) 3. U-Net | ResNet-18 | 512 px (0.9686)
0.80	≈0.033	1. U-Net | ResNet-18 | 256 px (0.9853) 2. UNet++ | ResNet-18 | 256 px (0.9770) 3. U-Net | ResNet-18 | 512 px (0.9641)
0.75	≈0.042	1. U-Net | ResNet-18 | 256 px (0.9833) 2. UNet++ | ResNet-18 | 256 px (0.9739) 3. U-Net | ResNet-18 | 512 px (0.9597)
0.70	0.050	1. U-Net | ResNet-18 | 256 px (0.9812) 2. UNet++ | ResNet-18 | 256 px (0.9707) 3. U-Net | ResNet-18 | 512 px (0.9552)

^a^ Calculated as (1 – mIoU Weight)/6.

## 3 Results

### 3.1 Cross-validation demonstrates high overall segmentation predictivity and moderate stability

The revised Bone-Pores (BP) segmentation model achieved consistently high predictivity across the 15 cross-validation runs. Mean IoU across five folds and three seeds was 0.9133 ± 0.0476 [mean Dice: 0.9492 ± 0.0318] (Table 6). Predictivity was highest in Test Fold 1, which consisted of scans from the river otter and mouse samples (Figures 3A,C,H, 5B); mean IoU was 0.9731 ± 0.0092 across seeds [mean Dice: 0.9863 ± 0.0047]. Test Folds 3 and 5 also showed good predictivity, with mean IoU values of 0.9393 ± 0.0367 [mean Dice: 0.9683 ± 0.0198] and 0.9267 ± 0.0972 [mean Dice: 0.9593 ± 0.0577], respectively. Test Folds 2 and 4 displayed greater variability related to the inclusion of scans with challenging morphology such as “AMNH_Mammals_M-89009_F” (Figures 1G, 4A) and “AMNH_Mammals_M-206440_mixed” (Figures 1I, 4B). Even so, mean IoU values across seeds remained above 0.85 (Supplementary Table S1) and above 0.90 when converted to mean Dice (Supplementary Table S2). Taken together, these results suggest that the scan-level partitioning removed the optimistic bias associated with slice-level data leakage in the previous BP-2D-02a model (Lee et al., 2025).

Because Test Fold 1 (seed 42) showed a good balance between high mean IoU (0.9731) and low variability (SD = 0.0099) [mean Dice: 0.9864 ± 0.0051], its training/validation pool was used as the baseline dataset for subsequent benchmarking experiments.

### 3.2 Reproducible performance across platforms

Fitting performance for the baseline model (BP-2D-03) was highly similar across the two high-end workstations and across both BONe implementations (Avizo and standalone). Validation mIoU ranged from 0.9736 to 0.9790 [Validation Dice: 0.9866–0.9894] across all combinations of workstation, operating system, BONe implementation, and GPU count (Table 4). Dual-GPU configurations reduced wall time substantially relative to single-GPU runs, although validation mIoU remained nearly identical. Implementations of “BONe DLFit” operating in Linux completed fitting substantially faster than their Windows counterparts, which likely stemmed from OS-level differences in GPU utilization (Table 4). Regardless of operating system, “BONe DLFit” produced nearly identical validation mIoU values.

Prediction performance of “BONE DLPred” was stable across platforms when applying model BP-2D-03 to scan “2R_2U_HF” from Test Fold 1 with a 2D chunk size of 512 px x 512 px (Table 5). Peak VRAM ranged from 12.0 GB on the low-end “Friday” workstation to 72.4 GB on “Jarvis” in dual-GPU mode. These differences reflect adaptive batch-size estimation during prediction, which scales the number of concurrently processed tiles to fit within the free GPU memory of each system. Both high- and low-end systems produced visually consistent segmentations (described below with “BONe IoU”), and wall times scaled predictably with hardware performance (Table 5).

“BONe IoU” was compared across platforms by comparing the predictions described in the preceding paragraph with the reference segmentation of scan “2R_2U_HF” from Test Fold 1. Identical IoU values were produced across platforms (Table 5). Put together, these results suggest that “BONe DLFit”, “BONe DLPred”, and “BONe IoU” are stable across a variety of computer configurations and behave reproducibly.

### 3.3 Effects of model architecture, backbone, and patch size

#### 3.3.1 U-Net and UNet++ showed the highest segmentation IoU

Across the 30 evaluated configurations, U-Net and UNet++ architectures consistently outperformed DeepLabV3+ and SegFormer (Supplementary Tables S3, S4). The top-performing U-Net and UNet++ models achieved mean IoU values of 0.9726–0.9740 [mean Dice: 0.9861–0.9868] with 256-px patches and simpler backbones (ResNet-18 or EfficientNet-B3). In contrast, the best-performing DeepLabV3+ and SegFormer models achieved mean IoU values of 0.9160 and 0.9174 [mean Dice: 0.9562–0.9569], respectively.

These findings indicate that architectures designed to preserve fine-scale spatial information through skip connections remain the most effective for distinguishing bone tissue vs. medullary pores in closely adjacent bony elements. Transformer-based models and atrous-convolution models benefited from a larger receptive window but did not match the fine-grained boundary detection achieved by U-Net and UNet++.

#### 3.3.2 Simpler backbones offered the best trade-off between mIoU and efficiency

ResNet-18 and EfficientNet-B3 backbones generally produced the strongest results across architectures (Supplementary Table S3). EfficientNet-B3 achieved among the highest mIoU values when paired with U-Net or UNet++. However, it consistently required substantially longer fitting times than other backbones, ironically related to less efficient use of available GPUs (Supplementary Table S3). In contrast, ResNet-18 provided high IoU with comparatively low computational cost, making it the most balanced backbone in terms of predictivity and efficiency. ResNet-50 increased parameter count and computational cost without consistently improving segmentation performance (Supplementary Table S3). MiT-B1 yielded competitive results but only when paired with U-Net and UNet++ architectures (Supplementary Table S3).

#### 3.3.3 Effects of patch size depended on architecture

Patch size influenced mIoU in architecture-specific ways. For U-Net and UNet++, 256-px patches consistently produced the highest mean IoU and the highest weighted scores. Increasing patch size to 512 px did not improve mIoU and resulted in substantial increases in VRAM usage, floating-point operations, and fitting time (Supplementary Table S3). These architectures appear to extract sufficient contextual information from smaller patches while maintaining sharp boundary localization.

In contrast, both DeepLabV3+ and SegFormer improved substantially when patch size increased from 256 px to 512 px. DeepLabV3+ models gained 0.02 to 0.07 mIoU [0.01–0.04 mDice], and SegFormer models gained approximately 0.02–0.04 mIoU [0.01–0.02 mDice] (Supplementary Tables S3S,S4). These increases reflect the importance of broader spatial context for models that rely on dilated convolutions or attention mechanisms. However, even with larger patches, neither architecture matched the segmentation performance achieved by U-Net or UNet++. Larger patches also incurred higher computational costs, especially for transformer-based models, which reduced their overall weighted scores and performance-efficiency ranking.

#### 3.3.4 Model rankings were stable under different performance-efficiency weighting schemes

Weighted scores were used to evaluate the joint effects of mIoU performance and computational efficiency. Across all weighting schemes tested, the U-Net architecture with ResNet-18 backbone and 256-px patch size (receptive window) remained the highest-ranked configuration or within the top three (Table 7). UNet++ with ResNet-18 also consistently ranked among the top models. Although the highest-mIoU model under a performance-only weighting was UNet++ with EfficientNet-B3 and 256-px patch size (Table 7), this configuration showed a combination of extremely long fitting time, high VRAM usage, and low GPU utilization (Supplementary Table S3) that substantially reduced its rank when efficiency metrics were included.

## 4 Discussion

This study introduces BP-2D-03 as the revised Bone-Pores segmentation model, replacing the earlier BP-2D-02a (Lee et al., 2025). The revised model was trained by an updated software pipeline that fixes key limitations of the previous workflow by removing slice-level data leakage, reducing memory demands, and supporting a larger and more varied dataset. These improvements allow the model to learn more stable and general features across diverse imaging conditions. Benchmarking experiments showed that the three parts of the software (“BONe DLFit”, “BONe DLPred”, and “BONe IoU”) perform reliably across a broad range of architectures, backbones, and patch sizes, and that results are stable across a variety of computer platforms. By evaluating 30 model combinations, we identified consistent strengths and limitations that translate into practical recommendations for users and clear directions for future development.

A notable outcome of the benchmarking experiments is the overall performance among architectures paired with convolution-based backbones (pattern-extractors or encoders). Both U-Net and UNet++ produced consistently high mean IoU values with relatively low variability, reflecting the well-documented strength of convolutional encoder–decoder design and skip connections in preserving fine spatial detail (Ronneberger et al., 2015; Zhou et al., 2018). Similar observations appear in recent hybrid convolution-transformer studies, which emphasize the importance of convolutional localization when improving transformer-based architectures (Chen et al., 2021; Tragakis et al., 2023). In contrast, transformer-based and atrous-convolution architectures such as SegFormer and DeepLabV3+ showed greater sensitivity to the spatial context provided by larger patches. This pattern is consistent with work demonstrating that transformer and dilated-convolution models benefit from wider receptive fields that capture long-range spatial dependencies (Chen et al., 2018; Xie et al., 2021). However, even with larger patches, these models did not consistently match the ability of U-Net or UNet++ to delineate high-resolution boundaries of bone and medullary pores. The analysis of backbones further supports this conclusion. ResNet-18 consistently provided a strong balance between predictivity and efficiency. In contrast, EfficientNet-B3, despite occasionally producing the highest mIoU scores, required substantially longer fitting times, likely caused by consistently low GPU utilization in our experiments. Although EfficientNet backbones achieve favorable theoretical FLOPs-to-segmentation trade-offs (Tan and Le, 2019), empirical efficiency depends strongly on hardware and software implementation (Prajwal et al., 2025), which may have contributed to the poor use of available GPUs observed here. Collectively, these results indicate that models with moderate architectural complexity and strong localization ability provide the best trade-off between quality and resource demands for segmentation of bone in micro-CT scans.

The weighted ranking framework jointly evaluates segmentation quality and computational efficiency, offering a more comprehensive assessment of model suitability than one based solely on performance. Across all weighting schemes, the U-Net with a ResNet-18 backbone and 256-px patches remained among the highest-ranked configurations, demonstrating that the ranking was robust to reasonable shifts in weighting emphasis. The performance-only emphasis identified UNet++ with EfficientNet-B3 and 256-px patches as the top model. However, its rank decreased substantially once fitting time, VRAM usage, and GPU utilization were considered. We chose a performance-to-efficiency weighting of 0.85:0.15 to reflect the primary importance of segmentation mIoU while recognizing that highly inefficient models are impractical for iterative experimentation. Long fitting times and poor GPU utilization reduce the feasibility of conducting replicate runs and limit scalability. These considerations align with a growing body of work arguing that model selection should balance predictivity with computational cost (e.g., Naser, 2023; Li et al., 2025; Prajwal et al., 2025). The ranking results therefore highlight the importance of evaluating model suitability not only in terms of mIoU but also in terms of the time and resources required to achieve that performance.

The cross-platform experiments demonstrated that BONe is robust to variation in operating system, hardware configuration, and implementation (Avizo 3D vs. standalone). In particular, “BONe DLFit” includes a reproducibility mode that produces bitwise-identical results when rerun on the same workstation, operating system, and implementation. This follows established recommendations for enforcing reproducible deep learning results by controlling random number generator states (e.g., in Python, NumPy, and PyTorch), enabling deterministic cuDNN GPU kernels, and deploying deterministic multiprocessing workers (e.g., Nagarajan et al., 2019; Chen et al., 2022; Heumos et al., 2023). However, as prior studies have shown, identical code and seeds cannot guarantee identical results across platforms because OS-level libraries and parallel execution can introduce small numerical differences (Glatard et al., 2015; Gundersen et al., 2022). We observed the same phenomenon, in which validation mIoU and mDice varied slightly across operating systems, implementations of “BONe DLFit”, and workstations (Table 4).

This phenomenon is specific to model fitting. Both “BONe DLPred” and “BONe IoU” perform inference and metric computation using algorithms that do not rely on randomness, GPU nondeterministic kernels, or parallel-reduction shortcuts known to introduce cross-platform variation (e.g., Chen et al., 2022; Gundersen et al., 2022; Heumos et al., 2023). As a result, these components produce exact, bitwise-identical outputs across repeated runs, and any observed differences in VRAM usage or wall time reflect hardware characteristics rather than algorithmic nondeterminism. Across experiments, segmentation outputs remained identical, and the shorter wall times observed in Linux likely reflect differences in GPU scheduling and background process management rather than BONe-specific behavior (Table 5). In summary, although “BONe DLFit” exhibits the well-documented sensitivity of deep learning training pipelines to underlying computational environments, the overall BONe workflow remains reliable and robust across different laboratory settings and is strictly reproducible under controlled ones.

The results also inform several directions for future development of the BONe software. The need to support both new and experienced users motivates the creation of two complementary interfaces. Although the current design philosophy is to limit the number of exposed hyperparameters, the interface can still be intimidating for beginners. An “Easy Mode” that automatically applies tested model settings and conservative defaults will help new users obtain strong results without navigating extensive configuration options. An “Advanced Mode” will expose even more parameters (e.g., model architecture, backbone, optimizer, scheduler, and augmentation settings) to better support exploratory or highly customized studies. The addition of customizable augmentation pipelines will allow users to tune image transforms and probabilities based on the variability and scale of their datasets. Expanding the set of available loss functions may help with datasets containing more complex segmentation classes. For example, a recent survey highlighted the robustness of the Focal Tversky loss function for segmentation tasks involving class imbalance or subtle boundaries (Azad et al., 2023). Additional optimizers and learning rate scheduling options may provide flexibility to tackle a broader range of segmentation tasks. Stochastic Gradient Descent (SGD) with momentum is an alternative optimizer that remains widely used in biomedical image segmentation because it often provides strong generalization and stable convergence when paired with an appropriate learning rate schedule (Nagendram et al., 2023). AdamW is another popular choice because it handles weight decay separately from gradient update and can reduce overfitting (Loshchilov and Hutter, 2019). Offering a multi-cycle option for cosine annealing (Loshchilov and Hutter, 2017) may support better exploration of the loss landscape.

The model fitting workloads indicate that our current multi-GPU implementation is limited by inter-GPU communication overhead. PyTorch’s “DataParallel” library offers a convenient mechanism for distributing batches across devices, but it centralizes gradient aggregation on a single GPU (PyTorch Contributors, 2025). This creates a communication bottleneck that caps scaling efficiency, which is evident in the reduced utilization observed in dual-GPU runs (Table 4). Transitioning to PyTorch’s Distributed Data Parallel (DDP), which distributes gradient synchronization across all GPUs (e.g., Aach et al., 2023), is expected to yield substantially better multi-GPU speedups. These gains matter most for model fitting because prediction workloads showed only minor improvements with two GPUs. Prediction involves only forward passes and lacks the gradient synchronization, so the overhead of splitting inputs and aggregating outputs across devices largely offsets any potential gains (Table 5). For this reason, efforts to integrate DDP will focus on improving fitting performance, where multi-GPU scaling has the greatest impact on wall time.

**FIGURE 9:**
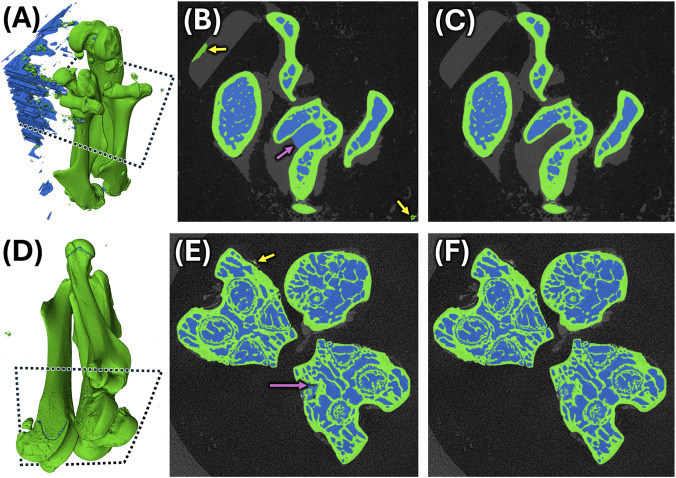
Performance of model BP-2D-03 on unseen validation and testing scans. **(A)** 3D view of predicted bone (green) and pores (blue) segmentation of validation scan “1R_1U_HF” with substantial mislabeled background (mIoU = 0.9593; mDice = 0.9792). Cross-sectional view of predicted segmentation **(B)** and corresponding reference **(C)** showing mislabeled background as bone (yellow arrows) and mislabeled pore overflowing into the intertrochanteric fossa of femur (magenta arrow). **(D)** 3D view of predicted segmentation of testing scan “2R_2U_HF” with few segmentation errors in the background (mIoU = 0.9829; mDice = 0.9914). Cross-sectional view of predicted segmentation **(E)** and corresponding reference **(F)** showing mislabeled background as bone (yellow arrow) and mislabeled growth plate pores as background (magenta arrow).

The cross-validation results provide insight into how dataset composition influences model robustness and highlight directions for future dataset design. Using the U-Net | ResNet-18 | 256-px patch configuration, model predictivity was high with a grand mIoU across folds and seeds exceeding 0.91 [mDice: 0.94]. Variability across random seeds was extremely small, indicating that the model fitting is not overly sensitive to stochastic differences in initialization or data shuffling. In contrast, performance varied more substantially across folds, reflecting heterogeneity in the underlying data rather than instability in the fitting procedure. Two test folds contained rare or challenging scans, namely, the composite scan (“AMNH_Mammals_M-89009_F”: Figures 1G, 4A) in Test Fold 2 and the low-resolution scan (“AMNH_Mammals_M-206440_mixed”: Figures 1I, 4B) in Test Fold 4. These observations suggest that although the model is stable overall, its performance can decrease for atypical or difficult scans in the validation pool or test fold (Figure 9). To guard against such outliers, future datasets should include multiple examples of each challenging type of scan to better capture the full spectrum of data variability.

The performance patterns observed here point to broader opportunities for incorporating additional spatial context into BONe models. Architectures that benefit from wider receptive fields, such as DeepLabV3+ and SegFormer, improved with larger 2D patches, which suggests that 2.5D representations may provide a more effective strategy for capturing cross-slice structure. Both implementations of BONe already support 2.5D models by using a number of adjacent slices as input to predict the center slice, and this shallow volumetric context may help reduce ambiguity with more complex segmentation tasks. Recent work further supports this direction. Avesta et al. (2023) found that 3D models provided the highest segmentation predictivity and maintained strong performance with limited training data, although they required 20 times more GPU memory than 2.5D and 2D approaches. However, other studies have shown that 3D models do not always outperform lower-dimensional alternatives. Crespi et al. (2022) and Zhang et al. (2022) reported cases in which 2.5D or 2D methods matched or exceeded 3D performance. Although the methods used in Lee et al. (2025) contained a data-leakage issue that has been addressed in the present study, the overall conclusion that 2D models can outperform 3D models under certain dataset and sampling conditions remains well supported. These findings indicate that the optimal dimensionality is strongly dependent on the characteristics of the imaging dataset. A systematic comparison of 2D, 2.5D, and 3D training strategies on the same dataset will therefore be an important direction for future development and is the focus of a dedicated follow-up study.

Taken together, the results of this benchmarking study establish BONe as a flexible, reproducible, and computationally efficient framework for micro-CT bone segmentation. The findings provide practical guidance for both new and advanced users. BONe supports two clear workflows. Users working with scans like those used in this study can apply the revised model BP-2D-03 directly. This workflow requires no additional training and only involves running “BONe DLPred” to generate segmentations and “BONe IoU” for optional quantitative evaluation. Users working with datasets that differ in imaging characteristics, anatomical structure, or noise profile can perform transfer learning. In this workflow, “BONe DLFit” is used to fine-tune BP-2D-03 (or another compatible pre-trained model) on a modest sample of representative scans, after which “BONe DLPred” and “BONe IoU” are used for deployment and evaluation. Either way, BONe offers a practical foundation for both routine analysis and methodological research in bone imaging.

## 5 Conclusion

This study introduces BONe, a flexible and reproducible deep learning software interface for segmenting bone and medullary pores in micro-CT scans. We evaluated its performance across a diverse set of architectures, backbones, patch sizes, and computational environments. By addressing the limitations of earlier workflows, including data leakage, memory inefficiency, and limited evaluation of model robustness, BONe provides a strong foundation for both routine segmentation and methodological research. The revised 2D model, BP-2D-03, offers strong predictivity across varied imaging conditions albeit with room for improvement. The software enables users to either deploy this model directly or fine-tune it to new datasets through transfer learning. Future developments, including expanded hyperparameter control, improved multi-GPU scaling, and systematic evaluation of 2D, 2.5D, and 3D approaches, will further enhance BONe’s flexibility. Collectively, these advances support scalable, reproducible, and high-quality bone segmentation for anatomical and biomedical applications.

## Data Availability

The datasets presented in this study can be found in online repositories. The names of the repository/repositories and accession number(s) can be found below: Publication link (https://doi.org/10.5061/dryad.4j0zpc8qq).
