# Supplementary material for: Deep learning software and revised 2D model to segment bone in micro-CT scans
Source: Front Bioinform. 2026 Jan 21;5:1677527. doi: 10.3389/fbinf.2025.1677527 (PMC12868216; doi:10.3389/fbinf.2025.1677527)
Supplement: Supplementary file 1 [file Table1.docx]

**Table S1**. Expanded results for Table 6. Detailed per-scan results for all 5-fold cross-validation folds and replicates. Each scan within a test fold was evaluated under three random seeds (42, 1701, 1864), with performance reported as mean Intersection over Union (mIoU). For each scan, the mean ± SD across seeds is provided as well as by the mean ± SD aggregated across scans within each fold.

| **Test Fold** | **Scan ID** | **mIoU**  **(seed 42)** | **mIoU**  **(seed 1701)** | **mIoU**  **(seed 1864)** | **Mean ± SD across seeds** |
| --- | --- | --- | --- | --- | --- |
| 1 | UF_Mammals_31151_HRU | 0.9745 | 0.9775 | 0.9726 | 0.9749 ± 0.0025 |
|  | OMNH_Mammals_44262_HRU | 0.9593 | 0.9587 | 0.9595 | 0.9591 ± 0.0004 |
|  | 2R_2U_HF | 0.9829 | 0.9832 | 0.9837 | 0.9833 ± 0.0004 |
|  | OMNH_Mammals_53994_HRU | 0.9758 | 0.9758 | 0.9736 | 0.9751 ± 0.0013 |
| Mean ± SD |  | 0.9731 ± 0.0099 | 0.9738 ± 0.0106 | 0.9723 ± 0.0099 | 0.9731 ± 0.0092 |
|  |  |  |  |  |  |
| 2 | UWBM_Mamm_81969_HRU | 0.9721 | 0.9726 | 0.9662 | 0.9703 ± 0.0036 |
|  | UWBM_Mamm_78743_FTFi | 0.9598 | 0.9503 | 0.9572 | 0.9558 ± 0.0049 |
|  | 12R_12U_HF | 0.9819 | 0.9795 | 0.9795 | 0.9803 ± 0.0014 |
|  | AMNH_Mammals_M-206440 | 0.4714 | 0.5107 | 0.5063 | 0.4961 ± 0.0215 |
| Mean ± SD |  | 0.8463 ± 0.2501 | 0.8533 ± 0.2287 | 0.8523 ± 0.2308 | 0.8506 ± 0.2142 |
|  |  |  |  |  |  |
| 3 | OMNH_Mammals:53994_FTFi | 0.9755 | 0.9726 | 0.9757 | 0.9746 ± 0.0017 |
|  | UWBM_Mamm:81969_FTFi | 0.9659 | 0.9664 | 0.9669 | 0.9664 ± 0.0005 |
|  | UF_Mammals_23593-24550_HF | 0.8922 | 0.8664 | 0.9201 | 0.8929 ± 0.0269 |
|  | UAM_Mam_67696_HF | 0.9347 | 0.9151 | 0.9199 | 0.9232 ± 0.0102 |
| Mean ± SD |  | 0.9420 ± 0.0375 | 0.9301 ± 0.0497 | 0.9456 ± 0.0298 | 0.9393 ± 0.0367 |
|  |  |  |  |  |  |
| 4 | 19R_19U_HF | 0.9551 | 0.9606 | 0.9225 | 0.9460 ± 0.0206 |
|  | 1R_1U_HF | 0.9450 | 0.9562 | 0.9073 | 0.9362 ± 0.0256 |
|  | AMNH_Mammals_M-89009_F | 0.6737 | 0.6687 | 0.5939 | 0.6454 ± 0.0447 |
|  | 7R_7U_HF | 0.9793 | 0.9800 | 0.9795 | 0.9796 ± 0.0004 |
| Mean ± SD |  | 0.8883 ± 0.1438 | 0.8913 ± 0.1488 | 0.8508 ± 0.1741 | 0.8768 ± 0.1425 |
|  |  |  |  |  |  |
| 5 | UAM_Mam_24789_FTFi | 0.9712 | 0.9703 | 0.9723 | 0.9712 ± 0.0010 |
|  | 5R_5U_HF | 0.9700 | 0.9704 | 0.9701 | 0.9701 ± 0.0002 |
|  | ZMB_Mam_30740_HRU | 0.8953 | 0.7858 | 0.6758 | 0.7856 ± 0.1098 |
|  | UAM_Mam_67696_TFiRU | 0.9804 | 0.9793 | 0.9799 | 0.9799 ± 0.0006 |
| Mean ± SD |  | 0.9542 ± 0.0396 | 0.9264 ± 0.0939 | 0.8995 ± 0.1492 | 0.9267 ± 0.0972 |
