# Supplementary material for: Deep learning software and revised 2D model to segment bone in micro-CT scans
Source: Front Bioinform. 2026 Jan 21;5:1677527. doi: 10.3389/fbinf.2025.1677527 (PMC12868216; doi:10.3389/fbinf.2025.1677527)
Supplement: Supplementary file 2 [file Table2.docx]

**Table S2**. Expanded results for Table 6 with mIoU converted to mDice. Detailed per-scan results for all 5-fold cross-validation folds and replicates. Each scan within a test fold was evaluated under three random seeds (42, 1701, 1864), with performance reported as mean Dice (mDice). For each scan, the mean ± SD across seeds is provided as well as by the mean ± SD aggregated across scans within each fold.

| **Test Fold** | **Scan ID** | **mDice**  **(seed 42)** | **mDice**  **(seed 1701)** | **mDice**  **(seed 1864)** | **Mean ± SD across seeds** |
| --- | --- | --- | --- | --- | --- |
| 1 | UF_Mammals_31151_HRU | 0.9871 | 0.9886 | 0.9861 | 0.9873 ± 0.0013 |
|  | OMNH_Mammals_44262_HRU | 0.9792 | 0.9789 | 0.9793 | 0.9792 ± 0.0002 |
|  | 2R_2U_HF | 0.9914 | 0.9915 | 0.9918 | 0.9916 ± 0.0002 |
|  | OMNH_Mammals_53994_HRU | 0.9878 | 0.9878 | 0.9866 | 0.9874 ± 0.0007 |
| Mean ± SD |  | 0.9864 ± 0.0051 | 0.9867 ± 0.0054 | 0.9860 ± 0.0051 | 0.9863 ± 0.0047 |
|  |  |  |  |  |  |
| 2 | UWBM_Mamm_81969_HRU | 0.9859 | 0.9861 | 0.9828 | 0.9849 ± 0.0018 |
|  | UWBM_Mamm_78743_FTFi | 0.9795 | 0.9745 | 0.9781 | 0.9774 ± 0.0026 |
|  | 12R_12U_HF | 0.9909 | 0.9896 | 0.9896 | 0.9901 ± 0.0007 |
|  | AMNH_Mammals_M-206440 | 0.6408 | 0.6761 | 0.6722 | 0.6630 ± 0.0194 |
| Mean ± SD |  | 0.8992 ± 0.1724 | 0.9066 ± 0.1538 | 0.9057 ± 0.1557 | 0.9038 ± 0.1455 |
|  |  |  |  |  |  |
| 3 | OMNH_Mammals:53994_FTFi | 0.9876 | 0.9861 | 0.9877 | 0.9871 ± 0.0009 |
|  | UWBM_Mamm:81969_FTFi | 0.9827 | 0.9829 | 0.9832 | 0.9829 ± 0.0003 |
|  | UF_Mammals_23593-24550_HF | 0.9430 | 0.9284 | 0.9584 | 0.9433 ± 0.0150 |
|  | UAM_Mam_67696_HF | 0.9662 | 0.9557 | 0.9583 | 0.9601 ± 0.0055 |
| Mean ± SD |  | 0.9699 ± 0.0201 | 0.9633 ± 0.0270 | 0.9719 ± 0.0158 | 0.9683 ± 0.0198 |
|  |  |  |  |  |  |
| 4 | 19R_19U_HF | 0.9770 | 0.9799 | 0.9597 | 0.9722 ± 0.0109 |
|  | 1R_1U_HF | 0.9717 | 0.9776 | 0.9514 | 0.9669 ± 0.0138 |
|  | AMNH_Mammals_M-89009_F | 0.8050 | 0.8015 | 0.7452 | 0.7839 ± 0.0336 |
|  | 7R_7U_HF | 0.9895 | 0.9899 | 0.9896 | 0.9897 ± 0.0002 |
| Mean ± SD |  | 0.9358 ± 0.0875 | 0.9372 ± 0.0907 | 0.9115 ± 0.1121 | 0.9282 ± 0.0889 |
|  |  |  |  |  |  |
| 5 | UAM_Mam_24789_FTFi | 0.9854 | 0.9849 | 0.9860 | 0.9854 ± 0.0005 |
|  | 5R_5U_HF | 0.9848 | 0.9850 | 0.9848 | 0.9849 ± 0.0001 |
|  | ZMB_Mam_30740_HRU | 0.9448 | 0.8801 | 0.8065 | 0.8771 ± 0.0692 |
|  | UAM_Mam_67696_TFiRU | 0.9901 | 0.9895 | 0.9898 | 0.9898 ± 0.0003 |
| Mean ± SD |  | 0.9763 ± 0.0211 | 0.9599 ± 0.0533 | 0.9418 ± 0.0902 | 0.9593 ± 0.0577 |
