# Supplementary material for: Deep learning software and revised 2D model to segment bone in micro-CT scans
Source: Front Bioinform. 2026 Jan 21;5:1677527. doi: 10.3389/fbinf.2025.1677527 (PMC12868216; doi:10.3389/fbinf.2025.1677527)
Supplement: Supplementary file 4 [file Table4.docx]

**Table S4**. Benchmarking 30 model configurations for Bone-Pores segmentation. Dataset used was Training/Validation Pool 1 with a random seed of 42. Results are the same as in Table S3 but IoU values are converted to Dice.

| **Model** | **Mean Dice** | **Bone Dice** | **Pores Dice** | **LR** | **Batch size** | **FLOPs (G)** | **Parameter count** | **Peak VRAM (GB)** | **Mean GPU use (%)** | **Fitting time (s)** | **Weighted score** |
| --- | --- | --- | --- | --- | --- | --- | --- | --- | --- | --- | --- |
| U-Net \| ResNet-18 \| 256 px | 0.9864 | 0.9882 | 0.9846 | 1e-3 | 64 | 21.33 | 14,322,227 | 22.51 | 83.02 | 8,996 | 0.9873 |
| U-Net \| ResNet-18 \| 512 px | 0.9856 | 0.9875 | 0.9837 | 1e-3 | 64 | 85.33 | 14,322,227 | 76.25 | 91.17 | 32,416 | 0.9686 |
| U-Net \| ResNet-50 \| 256 px | 0.9857 | 0.9878 | 0.9837 | 1e-3 | 64 | 42.36 | 32,515,123 | 38.42 | 88.91 | 12,868 | 0.9660 |
| U-Net \| ResNet-50 \| 512 px | 0.9854 | 0.9873 | 0.9834 | 1e-3 | 64 | 169.44 | 32,515,123 | 127.65 | 93.41 | 48,670 | 0.9435 |
| U-Net \| EfficientNet-B3 \| 256 px | 0.9868 | 0.9886 | 0.9849 | 1e-3 | 64 | 15.51 | 13,158,603 | 55.23 | 10.52 | 113,733 | 0.9588 |
| U-Net \| EfficientNet-B3 \| 512 px | 0.9855 | 0.9873 | 0.9837 | 1e-3 | 64 | 62.00 | 13,158,603 | 194.28 | 32.45 | 146,367 | 0.9257 |
| U-Net \| MiT-B1 \| 256 px | 0.9846 | 0.9855 | 0.9838 | 1e-3 | 64 | 20.02 | 16,426,163 | 32.13 | 16.54 | 53,256 | 0.9401 |
| U-Net \| MiT-B1 \| 512 px | 0.9834 | 0.9854 | 0.9814 | 1e-4 | 64 | 86.31 | 16,426,163 | 129.65 | 47.11 | 79,248 | 0.9166 |
| UNet++ \| ResNet-18 \| 256 px | 0.9861 | 0.9881 | 0.9842 | 1e-3 | 64 | 63.69 | 15,964,467 | 48.82 | 92.51 | 17,097 | 0.9802 |
| UNet++ \| ResNet-18 \| 512 px | 0.9854 | 0.9874 | 0.9834 | 1e-3 | 64 | 254.75 | 15,964,467 | 180.20 | 95.77 | 67,938 | 0.9443 |
| UNet++ \| ResNet-50 \| 256 px | 0.9853 | 0.9874 | 0.9832 | 1e-3 | 64 | 229.76 | 48,979,763 | 98.18 | 95.91 | 35,615 | 0.9365 |
| UNet++ \| ResNet-50 \| 512 px | 0.9857 | 0.9877 | 0.9837 | 1e-3 | 32 | 919.03 | 48,979,763 | 179.59 | 97.53 | 141,422 | 0.8769 |
| UNet++ \| EfficientNet-B3 \| 256 px | 0.9868 | 0.9886 | 0.9851 | 1e-3 | 64 | 31.02 | 13,624,363 | 60.98 | 14.35 | 119,508 | 0.9583 |
| UNet++ \| EfficientNet-B3 \| 512 px | 0.9856 | 0.9873 | 0.9838 | 1e-3 | 32 | 124.07 | 13,624,363 | 116.32 | 25.31 | 278,270 | 0.8962 |
| DeepLabV3+ \| ResNet-18 \| 256 px | 0.9281 | 0.9485 | 0.9068 | 1e-3 | 64 | 17.91 | 12,323,539 | 19.17 | 20.58 | 24,875 | 0.2878 |
| DeepLabV3+ \| ResNet-18 \| 512 px | 0.9548 | 0.9622 | 0.9474 | 1e-3 | 64 | 71.64 | 12,323,539 | 63.72 | 49.48 | 39,375 | 0.5909 |
| DeepLabV3+ \| ResNet-50 \| 256 px | 0.9443 | 0.9501 | 0.9384 | 1e-3 | 64 | 36.33 | 26,671,827 | 33.86 | 31.60 | 28,375 | 0.4607 |
| DeepLabV3+ \| ResNet-50 \| 512 px | 0.9562 | 0.9634 | 0.9489 | 1e-3 | 64 | 145.31 | 26,671,827 | 120.84 | 70.97 | 49,460 | 0.5919 |
| DeepLabV3+ \| EfficientNet-B3 \| 256 px | 0.9430 | 0.9490 | 0.9370 | 1e-3 | 64 | 12.06 | 11,679,979 | 58.63 | 11.90 | 93,133 | 0.4422 |
| DeepLabV3+ \| EfficientNet-B3 \| 512 px | 0.9557 | 0.9629 | 0.9484 | 1e-3 | 64 | 48.20 | 11,679,979 | 200.77 | 37.26 | 119,041 | 0.5725 |
| DeepLabV3+ \| MiT-B1 \| 256 px | 0.9132 | 0.9403 | 0.8848 | 1e-3 | 64 | 17.71 | 14,298,451 | 34.12 | 11.93 | 59,698 | 0.1168 |
| DeepLabV3+ \| MiT-B1 \| 512 px | 0.9514 | 0.9592 | 0.9435 | 1e-4 | 64 | 83.14 | 14,298,451 | 165.94 | 42.77 | 79,412 | 0.5298 |
| SegFormer \| ResNet-18 \| 256 px | 0.9420 | 0.9485 | 0.9353 | 1e-3 | 64 | 16.14 | 11,762,627 | 32.99 | 76.36 | 7,204 | 0.4591 |
| SegFormer \| ResNet-18 \| 512 px | 0.9556 | 0.9629 | 0.9482 | 1e-3 | 32 | 64.57 | 11,762,627 | 61.82 | 84.90 | 24,845 | 0.5868 |
| SegFormer \| ResNet-50 \| 256 px | 0.9425 | 0.9490 | 0.9359 | 1e-3 | 64 | 29.62 | 24,831,427 | 44.87 | 80.16 | 10,959 | 0.4553 |
| SegFormer \| ResNet-50 \| 512 px | 0.9569 | 0.9641 | 0.9496 | 1e-3 | 32 | 118.49 | 24,831,427 | 85.48 | 90.61 | 38,012 | 0.5892 |
| SegFormer \| EfficientNet-B3 \| 256 px | 0.9460 | 0.9524 | 0.9396 | 1e-3 | 64 | 11.37 | 11,189,595 | 59.20 | 8.68 | 112,022 | 0.4738 |
| SegFormer \| EfficientNet-B3 \| 512 px | 0.9568 | 0.9639 | 0.9494 | 1e-3 | 32 | 45.48 | 11,189,595 | 112.70 | 16.09 | 238,711 | 0.5550 |
| SegFormer \| MiT-B1 \| 256 px | 0.9342 | 0.9398 | 0.9285 | 1e-3 | 64 | 13.74 | 13,671,747 | 36.68 | 11.02 | 50,524 | 0.3474 |
| Segformer \| MiT-B1 \| 512 px | 0.9526 | 0.9603 | 0.9448 | 1e-4 | 32 | 61.20 | 13,671,747 | 78.20 | 23.19 | 111,927 | 0.5232 |

Abbreviations: LR=learning rate
